# The impact of time factors on overall survival in patients with nasopharyngeal carcinoma: a population-based study

**DOI:** 10.1186/s13014-016-0638-2

**Published:** 2016-04-27

**Authors:** Po-Chun Chen, Wen-Shan Liu, Wei-Lun Huang, Cheng-Jung Wu, Ching-Chieh Yang, Ching-Chih Lee

**Affiliations:** Department of Radiation oncology, Pingtung Christian Hospital, Pingtung, Taiwan; Graduate Institute of Bioresources, National PingTung University of Science and Technology, Pingtung, Taiwan; Department of Radiation oncology, Kaohsiung Veterans General Hospital, Kaohsiung, Taiwan; School of Medicine, National Defense Medical Center, Taipei, Taiwan; Department of Otolaryngology, Shung Ho Hospital, Taipei Medical University, Taipei, Taiwan; Department of Radiation oncology, Chi-Mei Medical Center, Tainan, Taiwan; Department of Otolaryngology, Kaohsing Veterans General Hospital, No.386, Dazhong 1st Rd., Kaohsiung, Taiwan

**Keywords:** Nasopharyngeal carcinoma, Time factors, Survival, Treatment outcome

## Abstract

**Background:**

Nasopharyngeal carcinoma (NPC) is most common in Southeast Asia. The purpose of this study is to investigate the correlation between wait time and length of radiotherapy and overall survival (OS) of NPC patients in Taiwan.

**Methods:**

From Taiwan’s National Health Insurance Research Database, this nationwide population-based cohort study identified 3605 NPC patients who received radiotherapy between 2008 and 2011. The impact of time factors on NPC survival rates was estimated with Kaplan-Meier survival curves. A multivariable Cox hazards regression model tested the significance of results after adjustment for other covariables.

**Results:**

In all, 317 patients had wait times >4 weeks, 1404 patients had longer duration of radiotherapy (i.e., >10 weeks) and 499 died. Patients with wait times > 4 weeks and length of radiotherapy ≤ 10 weeks didn’t have significantly inferior survival. Patients with wait times >4 weeks and length of radiotherapy >10 weeks had significantly lower OS than other groups, with an adjusted hazard ratio of 1.7 (95 % CI, 1.10–2.60).

**Conclusion:**

Time was a significant prognostic factor for NPC patients who had both >4 weeks wait times and length of radiotherapy >10 weeks. Patients with wait time > 4 weeks and length of radiotherapy ≤ 10 weeks had a trend toward an inferior survival.

## Introduction

Nasopharyngeal carcinoma (NPC) is an endemic malignant disease in Southeast Asia, but uncommon in Western countries [1]. The approximately 1504 cases of NPC diagnosed in Taiwan in 2009 accounted for 1.73 % of all cancer patients that year. The non-viral risk factors include family history, history of chronic rhinitis, consumption of salted fish, alcohol consumption, herbal product use, smoking and occupation exposure [2]. The possible associations, including smoking, Epstein-Barr virus activation and risk of NPC, have been published recently [3]. The standard of care is radiotherapy with or without adjuvant chemotherapy [4, 5].

There is an increasing interest in associations of treatment delays, duration of radiotherapy and outcomes in head-and-neck cancers. In general, treatment delay may be related to disease progression. The long treatment duration of radiotherapy may also contribute to poor tumor control by facilitating tumor repopulation. A 2008 review article summarized the many findings that treatment delay may be related to poor local control and decreased survival in head-and-neck cancers [6]. In contrast, a Dutch study found better disease-specific survival rates in head-and-neck squamous cell carcinoma patients with a delay of more than 30 days than in those treated within 30 days [7]. These studies mostly focused on head-and-neck carcinoma. Moreover, duration of radiotherapy more than eight weeks may influence the results in NPC [8]. Current evidence suggests that time factors influence treatment outcomes. However, to date, most studies have investigated only treatment delays (i.e., wait times) or duration of radiotherapy individually and few have looked at these in NPC. The potentially synergistic impact on survival in NPC patients of treatment delay and longer duration of radiotherapy is not well established.

We use the nationwide claims data from Taiwan’s National Health Insurance (NHI) research database to identify NPC patients treated with radiotherapy alone or with concurrent chemoradiotherapy between 2008 and 2011. This database provides basic demographic data as well as hospital characteristics, patient characteristics, and treatment modality. By determining the effect of treatment delay and longer course of radiotherapy, we hope to help healthcare providers better understand this patient population and the impact of time factors on survival.

## Patients and methods

### Ethical consideration

This study was approved by the Institutional Review Boards of Kaohsiung Veterans General Hospital and Buddhist Dalin Tzu Chi General Hospital, Taiwan. Review board requirements for written informed consent were waived because all personal identifying information was removed from the dataset prior to analysis.

### Study population

**Fig. 1:**
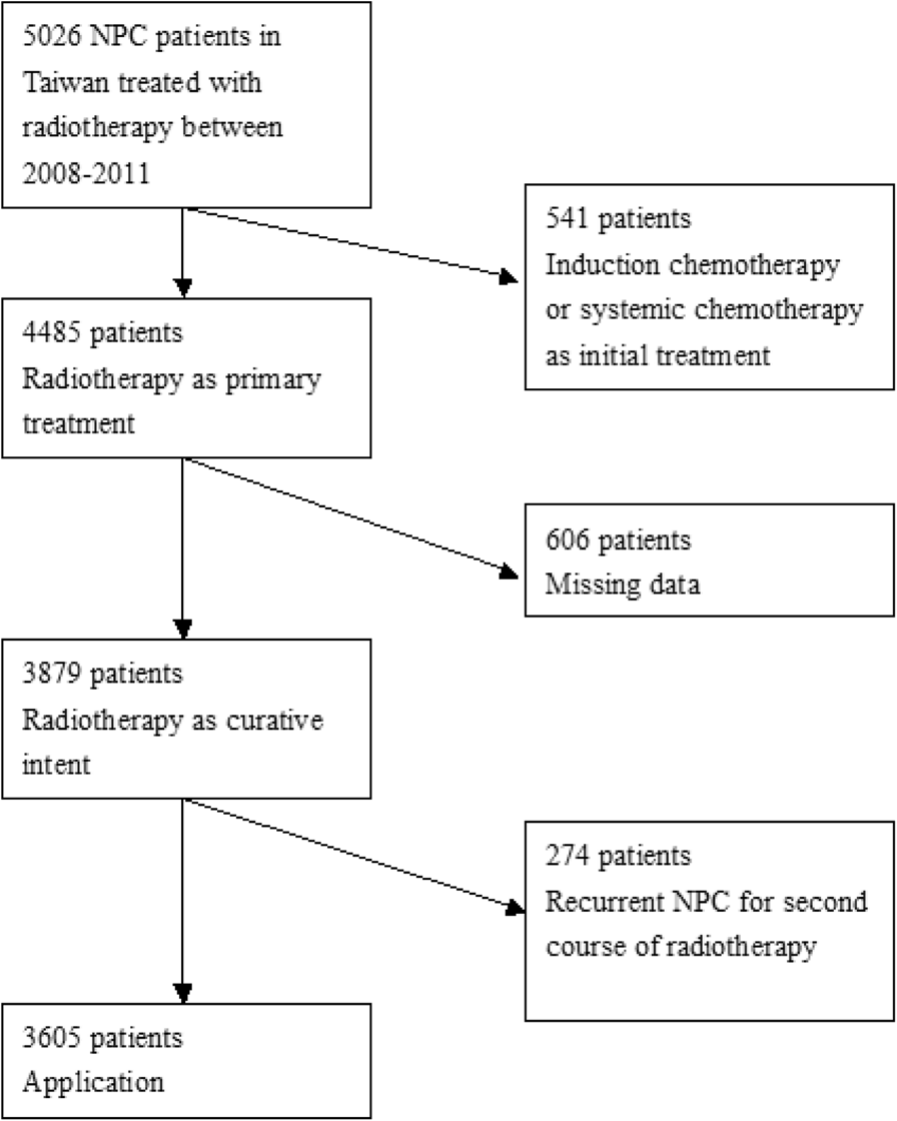
Flowchart of patient selection

The Taiwan NHI Research Database covered 92 % of the population in 1995 and 99 % after 2003. From this data, we identified 5026 NPC patients treated with radiotherapy from 2008 to 2011. We excluded patients who received induction or systemic chemotherapy as the initial treatment except for patients who received chemotherapy within 14 days prior to radiotherapy. Concurrent chemoradiotherapy (CCRT) is a standard treatment for NPC, but radiotherapy is often delayed to allow patient preparation and machines scheduling. Thus, we assumed the above condition as CCRT in this study. We also excluded patients who were treated for a second course of radiotherapy (Fig. 1). This left 3605 patients who matched the inclusion criteria for this study. From the Universal Deaths Database, we obtained patient death information for the same patient cohort.

### Wait times and length of treatment

Wait time was calculated from the date of diagnosis to the first day of radiotherapy. We hypothesized that wait times longer than 4 weeks would be associated with reduced overall survival. Length of treatment was calculated from the first day of radiotherapy to the last day of radiotherapy. Previous studies found that treatment lengths of seven, eight, or nine weeks influence the results in head-and-neck cancers [8, 9]. We used the cutpoint of >10 weeks to define prolonged length of radiotherapy.

### Other covariables

Basic data included hospital characteristics (ownership and teaching level), patient gender and age, treatment modality, urbanization and region of patient residence and Charlson Comorbidity Index Score (CCIS). Severity of comorbidity was based on the modified CCIS recorded before the diagnosis of NPC. The CCIS is a widely accepted scale used for risk adjustment in administrative claims data sets [10]. A meta-analysis demostrated that comorbidity was also a prognostic factor in NPC outcomes [11].

### Statistical analysis

The primary independent varialbes in our study were wait time and length of radiotherapy. Death in NPC patients was the event of interest. The Chi-squared test was used to compare the frequency of categorical independent variables. The impact of time factors on NPC survival rates was estimated with Kaplan-Meier survival curves, and differences were assessed by means of the log-rank statistic. A multivariable Cox hazards regression model was used to estimate hazard ratios (HRs) with 95 % confidence intervals (CIs) of death from any cause associated with wait time and length of radiotherapy. All statistical operations were performed using SPSS software (version 15, SPSS Inc., Chicago, IL). A two-tailed value of *p* < 0.05 was considered a significant difference.

## Results

**Fig. 2:**
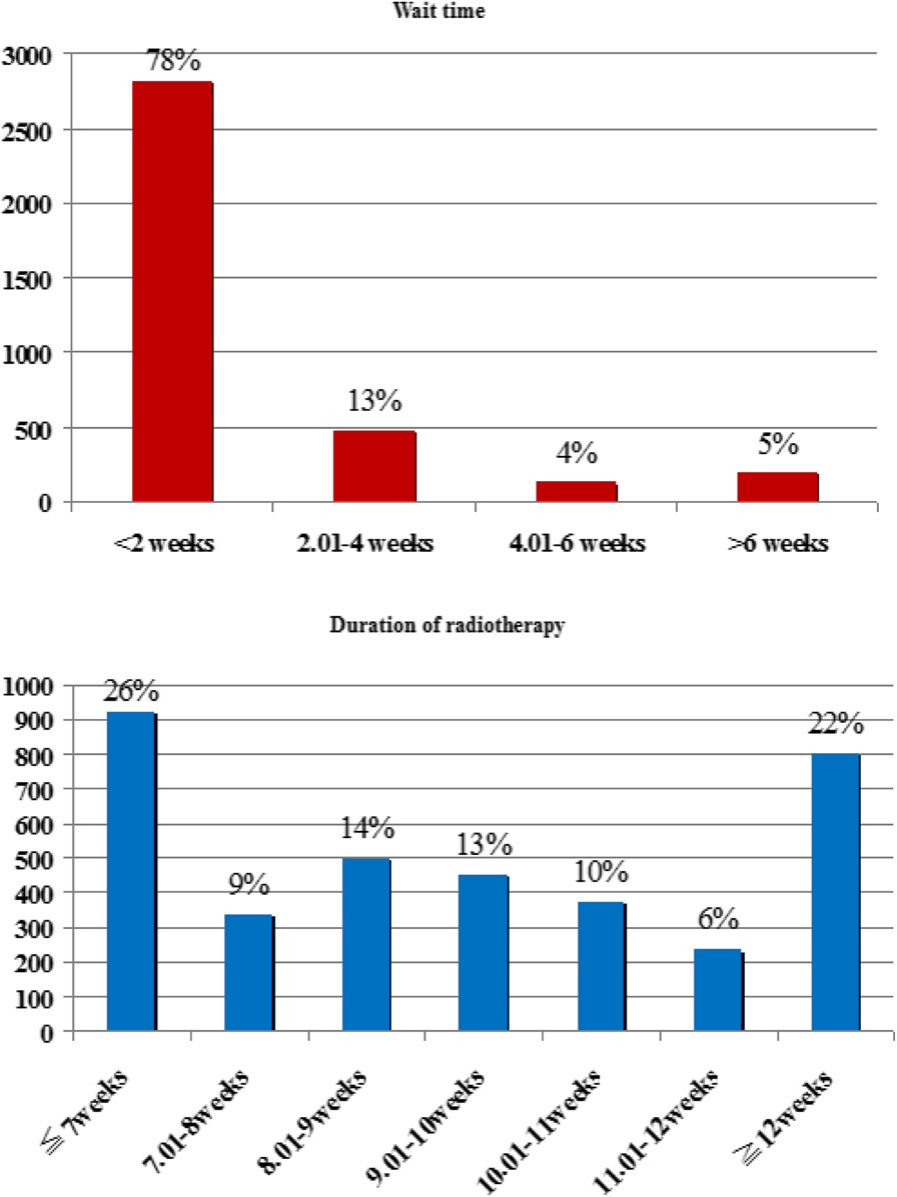
The distribution of wait time and duration of radiotherapy

The study identified a total of 3605 NPC patients treated with radiotherapy alone or CCRT from 2008 to 2011. Table 1 shows the demographic data for patients and hospitals. Most patients were male (75.2 %) and were treated at medical center (64.7 %). Most, or 3155 patients, were younger than 65 years (87.5 %). The majority of patients received CCRT (82.4 %). Approximately 70 % of patients had lower CCIS. Three hundred and seventeen patients (8.8 %) had a wait time >4 weeks and 1404 (38.9 %) patients had a course of radiotherapy lasting >10 weeks. Figure 2 showed the distribution of wait time and duration of radiotherapy.

**Table 1:** Hospital and demographic characteristics of nasopharyngeal cancer patients treated from 2008 to 2011 (*n* = 3605)

	Wait time				*P* value	Length of radiotherapy				*P* value
	Less than 4 weeks (*N* = 3288)		More than 4 weeks (*N* = 317)			Less than 10 weeks (*N* = 2201)		More than 10 weeks (*N* = 1404)		
	No.	%	N	%		No.	%	N	%	
Hospital characteristics					0.008					<0.001
Ownership										
For Profit (*n* = 1915)	1770	53.8	145	45.7		1114	50.6	801	57.1	
Non-profit (*n* = 563)	498	15.1	65	20.5		396	18.0	167	11.9	
Public (*n* = 1127)	1020	31.1	107	33.8		691	31.4	436	31.1	
Teaching level					0.442					0.006
Medical center (*n* = 2332)	2131	64.8	201	63.4		1394	63.3	938	66.8	
Regional (*n* = 1121)	1015	30.9	106	33.4		724	32.9	397	28.3	
District (*n* = 152)	142	4.3	10	3.2		83	3.8	69	4.9	
Patient characteristics										
Gender					0.498					0.151
Male (*n* = 2711)	2472	75.2	239	75.4		1637	74.4	1074	76.5	
Female (*n* = 894)	816	24.8	78	24.6		564	25.6	330	23.5	
Age group					<0.001					0.185
0–44.99 years (*n* = 1239)	1166	35.5	73	23.0		733	33.3	506	36.0	
45–64.99 years (*n* = 1916)	1739	52.9	177	55.8		1182	53.7	734	52.3	
More than 65 years (*n* = 450)	383	11.6	67	21.1		286	13.0	164	11.7	
Treatment					<0.001					<0.001
CCRT^a^ (*n* = 2970)	2789	84.8	181	57.1		1705	77.5	1265	90.1	
RT^b^ alone (*n* = 635)	499	15.2	136	42.9		496	22.5	139	9.9	
Charlson comorbidity index score (mean = 0.3778)					<0.001					0.053
Lower than mean (*n* = 2597)	2411	73.3	186	58.7		1611	73.2	986	70.2	
Higher than mean (*n* = 1008)	877	26.7	131	41.3		590	26.8	418	29.8	
Urbanization of resdence					0.045					0.728
Urban (*n* = 1149)	1056	32.1	93	29.3		692	31.4	457	32.5	
Suburban (*n* = 1508)	1386	42.2	122	38.5		922	41.9	586	41.7	
Rural (*n* = 948)	846	25.7	102	32.2		587	26.7	361	25.8	
Geographic region					<0.001					0.021
Northern (*n* = 1745)	1745	53.1	133	42.0		1122	51.0	756	53.8	
Central (*n* = 502)	502	15.3	53	16.7		370	16.8	185	13.2	
Southern (*n* = 946)	946	28.8	113	35.6		636	28.9	423	30.1	
Eastern (*n* = 95)	95	2.9	18	5.7		73	3.3	40	2.8	

^a^ *CCRT* concurrent chemoradiotherapy, ^b^ *RT* radiotherapy

**Fig. 3:**
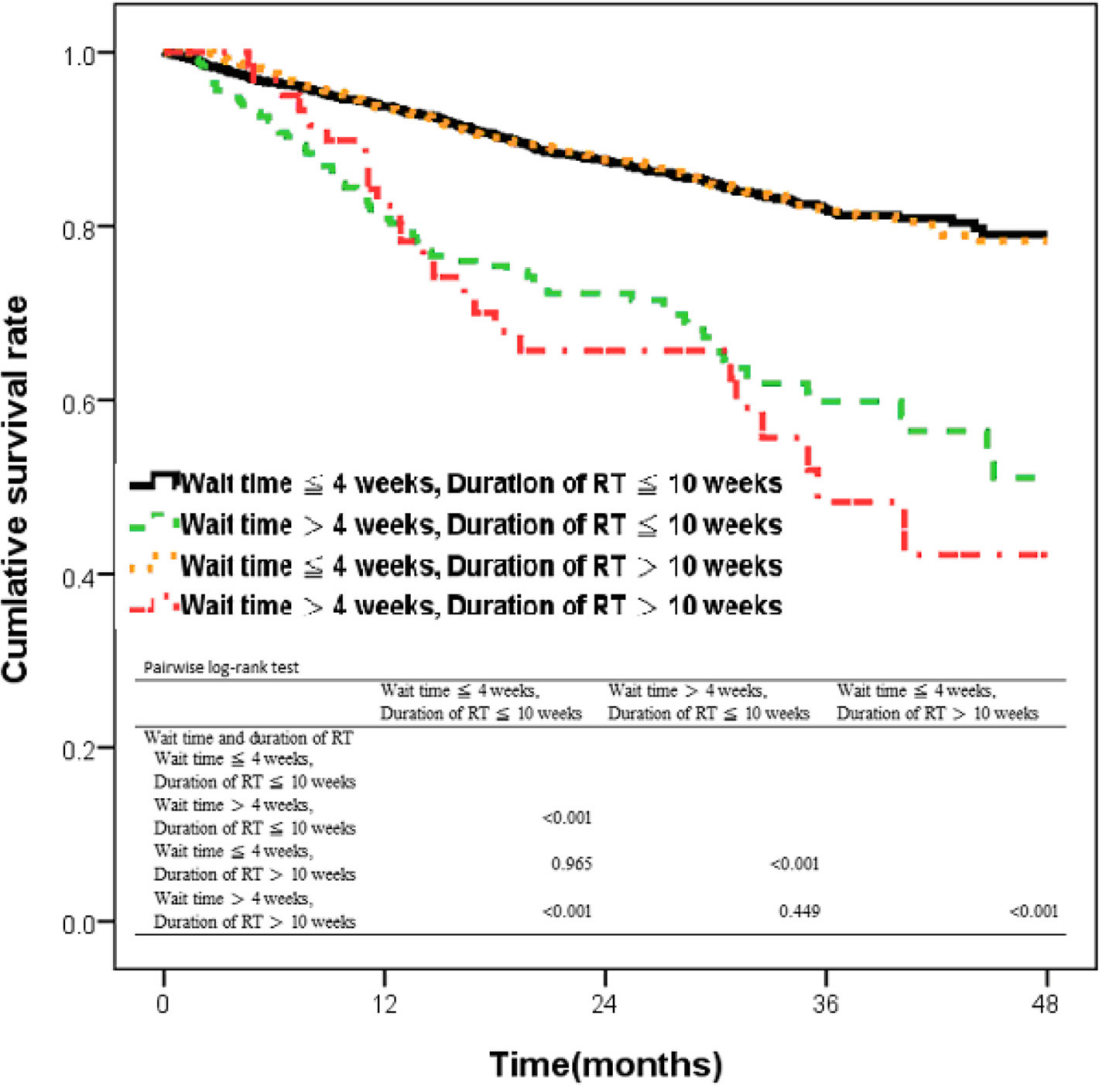
Kaplan-Meier Estimates of overall survival

A total of 499 deaths were identified in this cohort. Wait time combined with length of radiotherapy was categorized into four groups: wait time ≤4 weeks and length of radiotherapy ≤10 weeks; wait time >4 weeks and length of radiotherapy ≤10 weeks; wait time ≤4 weeks and length of radiotherapy >10 weeks; and wait time >4 weeks and length of radiotherapy >10 weeks. Kaplan-Meier curve revealed patients with wait time >4 weeks and length of radiotherapy ≤10 weeks, or wait time >4 weeks and length of radiotherapy >10 weeks had lower rate of overall survival than other groups (*P* < 0.001 by the log rank test) (Fig. 3).

Multivariable Cox hazards regression analyses revealed that patients who had wait times >4 weeks and length of radiotherapy ≤10 weeks had survival rates not signicant different from those of patients with wait times ≤4 weeks and length of radiotherapy ≤10 weeks. Patients with wait times >4 weeks and length of radiotherapy >10 weeks had significantly lower OS than those with shorter time factors, with an adjusted HR of 1.7 (95 % CI: 1.10–2.60) (Table 2). Patients older than 65 years, those with high CCIS and those who received radiotherapy alone had significantly lower survival rate (*P* < 0.001). Hospital ownership, hospital teaching level, gender and urbinization were not significantly associated with overall survival.

**Table 2:** Multivariate adjusted hazard ratio for death in nasopharyngeal cancer patients (*n* = 3605)

	Events/total (%)	Adjusted hazard ratio^d^	95 % CI^c^	*p* value
Wait time and duration of RT^b^				0.053
Wait time ≤ 4 weeks, duration of RT ≤ 10 weeks	223/1946 (11.5)	1		
Wait time > 4 weeks, duration of RT ≤ 10 weeks	75/255 (29.4)	1.26	(0.95–1.67)	0.097
Wait time ≤ 4 weeks, duration of RT > 10 weeks	177/1342 (13.2)	1.04	(0.85–1.27)	0.683
Wait time > 4 weeks, duration of RT > 10 weeks	24/62 (38.7)	1.70	(1.10–2.60)	0.015
Hospital ownership				0.183
For profit	135/1127 (12.0)	1		
Non-profit	273/1915 (14.3)	1.13	(0.91–1.40)	0.238
Public	91/563 (16.2)	1.28	(0.98–1.67)	0.069
Hospital teaching level				0.552
Medical center	313/2332 (13.4)	1		
Regional	162/1121 (14.5)	1.11	(0.91–1.36)	0.278
District	24/152 (15.8)	1.01	(0.66–1.55)	0.948
Gender				
Male	387/2711 (14.3)	1		
Female	112/894 (12.5)	0.98	(0.79–1.22)	0.900
Age group				<0.001
0–44.99 years	108/1239 (8.7)	1		
45–64.99 years	240/1916 (12.5)	1.25	(0.99–1.58)	0.052
More than 65 years	151/450 (33.6)	2.65	(2.01–3.49)	<0.001
Treatment				
CCRT^a^	356/2970 (12.0)	1		
RT^b^ alone	143/635 (22.5)	1.58	(1.26–1.98)	<0.001
Charlson Comorbidity Index Score (mean = 0.3778)				
Lower than mean	104/2597 (4.0)	1		
Higher than mean	395/1008 (39.2)	15.34	(12.26–19.18)	<0.001
Urbanization				0.598
Urban	138/1149 (12.0)	1		
Suburban	198/1508 (13.1)	0.90	(0.72–1.12)	0.374
Rural	163/948 (17.2)	0.89	(0.70–1.13)	0.372

^a^ *CCRT* concurrent chemoradiotherapy, ^b^ *RT*, radiotherapy

^c^95 % CI, 95 % confidence interval

^d^Adjusted for patient age, wait time, length of radiotherapy, gender, treatment, urbanization, Charlson Comorbidity Index Score, and hospital characteristics

## Discussion

In this study, we found a significant negative impact on survival of time factors only for patients with both prolonged wait times and a prolonged course of radiotherapy. Neither of these adverse factors alone had a significant impact on survival. In others words, if treatment delay can not be avoided because of patient factors, treatment schedules or health policy, healthcare providers should attempt to minimize the radiotherapy treatment time. Hyperfractionated radiotherapy may be considered and would be expected to reduce the interval by approximately one week (i.e., 74.4 and 31 treatment days; 78 Gy and 30 treatment days) [12–14]. Although some institutions prefer to schedule CCRT followed by adjuvant chemotherapy if chemotherapy is indicated, a strategy to reduce delays in radiotherapy might consider scheduling neoadjuvant chemotherapy after CCRT [15].

This study has three main strengths. First, NPC is endemic in Taiwan, which permits the collection of a large sample size for valid estimates. Patients are followed in the NHI research database and deaths recorded in the Univeral Deaths Database. Second, the NHI research database captures complete follow-up information of the comprehensive health care with a moderate cost sharing, and records any treatment. It therefore provides an accurate record of NPC rates and treatment in Taiwan. Validation of the NHI research database is regularly evaluated via comparison of chart-based records and claims-based records [16]. Third, the percentage of NPC patients experiencing treatment delays and longer durations of radiotherapy was 8.8 and 38.9 %, respectively. The significance of these clinical observations is worthy of further investigation.

Little evidence has been found to suggest correlations between treatment delays and outcomes for NPC patients. While some studies have investigated the impact of treatment delays on outcome (i.e., local control, survival) for head-and-neck cancer patients treated with primary radiotherapy, their results are controversial. Delaying radiotherapy (>40 days) has a deleterious effect on local control and overall survival in early stage head-and-neck squamous cell carcinoma [17]. To our knowledge, only one published study has shown a negative impact on survival of treatment delay for unresected head-and neck carcinoma patients. Another study found no effect of delay in the initiation of primary radiotherapy on disease-specific survival in patients with head-and-neck carcinoma [18]. Three studies evaluated the impact of treatment delay on local control [19–21]. No negative effect of treatment delay was demonstrated for laryngeal carcinoma or T1 nasopharyngeal carcinoma. A recent study investigated the association of treatment delay and prognosis in head-and-neck carcinoma [7]. The study examined 2493 patients treated with primary radiotherapy or adjuvant radiotherapy. After adjustment for age, site, stage, and treatment modality, multivariate regression models revealed that patients with a delay of >30 days had a better disease specific survival than those with no delay, and those with a delay of up to 90 days had no impaired survival.

Some studies have found that treatment time affects outcomes in patients with head-and-neck cancer. Sher et al. [22] analyzed the TAX-324 study to demonstrate that prolonged radiation treatment time was independently predictive of significantly inferior overall survival and local-regional control rate in head-and-neck cancer. Moreover, the locoregional failure rate increased by 3.3 % for each day of interruption of radiothrapy, and interruptions of more than 7 days were associated with an 18 % reduction in 5-year survival rates [8, 23]. However, we found that longer treatment times (i.e., >10 weeks) significantly affected survival only for patients who also had longer wait times (>4 weeks, HR = 1.70, *p* = 0.015). Though we could not know the exact reason of this finding, however, it could be explained that the total duration from diagnosis to the end of radiotherapy was more important than any single factor of waiting time or treatment time.

Our series has several limitations. First, cancer stage was not obtained. In order to exclude patients with distant metastasis, we excluded those patients whose initial chemotherapy and radiotherapy were separated by more than two weeks. This procedure also excluded patients who received induction chemotherapy. Second, we did not know the reasons why patients received radiotherapy alone or CCRT.

## Conclusion

Radiotherapy is still the main treatment for NPC patients. Patients in some countries may have longer wait times due to limited availability of radiotherapy facilities or health policy standards. Our study found that time is a significant prognostic factor for NPC patients only among those whose wait time was more than 4 weeks and whose radiotherapy lasted more than 10 weeks. Patients with wait time > 4 weeks and length of radiotherapy ≤ 10 weeks had a trend toward an inferior survival. Future investigations should also examine stage, radiation dose, and the types of chemotherapeutic drugs used.

## References

[1] Wei WI, Sham JS (2005). Nasopharyngeal carcinoma. Lancet.

[2] Jia WH, Qin HD (2012). Non-viral environmental risk factors for nasopharyngeal carcinoma: a systematic review. Semin Cancer Biol.

[3] Xu FH, Xiong D, Xu YF (2012). An epidemiological and molecular study of the relationship between smoking, risk of nasopharyngeal carcinoma, and Epstein-Barr virus activation. J Natl Cancer Inst.

[4] Chen QY, Wen YF, Guo L (2011). Concurrent chemoradiotherapy vs radiotherapy alone in stage II nasopharyngeal carcinoma: phase III randomized trial. J Natl Cancer Inst.

[5] Zhang L, Zhao C, Ghimire B, et al. The role of concurrent chemoradiotherapy in the treatment of locoregionally advanced nasopharyngeal carcinoma among endemic population: a meta-analysis of the phase III randomized trials. BMC Cancer. 2010;10:558. Available from URL: http://www.biomedcentral.com/1471-2407/10/558. [Accessed 24 Mar 2014]. 10.1186/1471-2407-10-558 PMC2970609 20950416

[6] Chen Z, King W, Pearcey R, Kerba M, Mackillop WJ (2008). The relationship between waiting time for radiotherapy and clinical outcomes: a systematic review of the literature. Radiother Oncol.

[7] van Harten MC, de Ridder M, Hamming-Vrieze O, Smeele LE, Balm AJ, van den Brekel MW (2014). The association of treatment delay and prognosis in head and neck squamous cell carcinoma (HNSCC) patients in a Dutch comprehensive cancer center. Oral Oncol.

[8] Kwong DL, Sham JS, Chua DT, Choy DT, Au GK, Wu PM (1997). The effect of interruptions and prolonged treatment time in radiotherapy for nasopharyngeal carcinoma. Int J Radiat Oncol Biol Phys.

[9] Cannon DM, Geye HM, Hartig GK, et al. Increased local failure risk with prolonged radiation treatment time in head and neck cancer treated with concurrent chemotherapy. Head Neck. 2013. Available from URL: http://onlinelibrary.wiley.com/doi/10.1002/hed.23419/abstract. [Accessed 24 Mar 2014]. 10.1002/hed.23419 23804248

[10] Deyo RA, Cherkin DC, Ciol MA (1992). Adapting a clinical comorbidity index for use with ICD-9-CM administrative databases. J Clin Epidemiol.

[11] Boje CR (2014). Impact of comorbidity on treatment outcome in head and neck squamous cell carcinoma - A systematic review. Radiother Oncol.

[12] Jian JJ, Cheng SH, Tsai SY (2002). Improvement of local control of T3 and T4 nasopharyngeal carcinoma by hyperfractionated radiotherapy and concomitant chemotherapy. Int J Radiat Oncol Biol Phys.

[13] He XY, Liu TF, He SQ, Huan SL, Pan ZQ (2007). Late course accelerated hyperfractionated radiotherapy of nasopharyngeal carcinoma (LCAF). Radiother Oncol.

[14] Pan ZQ, He XY, Guo XM (2012). A phase III study of late course accelerated hyperfractionated radiotherapy versus conventionally fractionated radiotherapy in patients with nasopharyngeal carcinoma. Am J Clin Oncol.

[15] Langendijk JA, Leemans CR, Buter J, Berkhof J, Slotman BJ (2004). The additional value of chemotherapy to radiotherapy in locally advanced nasopharyngeal carcinoma: a meta-analysis of the published literature. J Clin Oncol.

[16] Cheng CL, Kao YH, Lin SJ, Lee CH, Lai ML (2011). Validation of the National Health Insurance Research Database with ischemic stroke cases in Taiwan. Pharmacoepidemiol Drug Saf.

[17] Fortin A, Bairati I, Albert M, Moore L, Allard J, Couture C (2002). Effect of treatment delay on outcome of patients with early-stage head-and-neck carcinoma receiving radical radiotherapy. Int J Radiat Oncol Biol Phys.

[18] Leon X, de Vega M, Orús C, Morán J, Vergés J, Quer M (2003). The effect of waiting time on local control and survival in head and neck carcinoma patients treated with radiotherapy. Radiother Oncol.

[19] Lee AW, Chan DK, Fowler JF (1994). T1 nasopharyngeal carcinoma: the effect of waiting time on tumor control. Int J Radiat Oncol Biol Phys.

[20] Barton MB, Morgan G, Smee R, Tiver KW, Hamilton C, Gebski V (1997). Does waiting time affect the outcome of larynx cancer treated by radiotherapy?. Radiother Oncol.

[21] Brouha XD, Op De Coul B, Terhaard CH, Hordijk GJ (2000). Does waiting time for radiotherapy affect local control of T1N0M0 glottic laryngeal carcinoma?. Clin Otolaryngol Allied Sci.

[22] Sher DJ, Posner MR, Tishler RB (2011). Relationship between radiation treatment time and overall survival after induction chemotherapy for locally advanced head-and-neck carcinoma: a subset analysis of TAX 324. Int J Radiat Oncol Biol Phys.

[23] Xu L, Pan J, Wu J (2010). Factors associated with overall survival in 1706 patients with nasopharyngeal carcinoma: significance of intensive neoadjuvant chemotherapy and radiation break. Radiother Oncol.

